# The Beginnings of Disease

**Published:** 1893-02-04

**Authors:** 


### The Hospital

An Institutional Journal of

Science, Medicine, Nursing, and Philanthropy.

*For Hospitals, Asylums, Infirmaries, Dispensaries, Medical Practitioners, Students, Nurses, and the Charitable Public.*

Vol. XIII.—No. 332.] [Feb. 4, 1893.

### THE BEGINNINGS OF DISEASE.

The physician who has not something of the priest in him is generally a sorry fellow. We do not love priests in this country, because we misunderstand, and sometimes wilfully, both them and their function. The true priest is the fatherly shepherd of his flock, and the true physician is in like manner the fatherly shepherd of his flock from a different point of view. Dr. Campbell Pope publishes an address on "The Beginnings of Disease," which from the first line to the last expresses the longings of a large paternal heart for the physical, and inclusively for the mental and moral happiness of that large class of men, women, and children who need medical shepherding and care. Dr. Pope is not a mere medical priest and father: he is a trained man of science, and of large and careful observation.

Speaking of the daily work of the family practitioner of medicine, this thoughtful student of the ways of the sick and the well, says: "The nature of our labours, whilst not allowing us opportunity to study minutely the diseases of special organs, gives us this advantage over the specialist, that we are enabled to have an outlook over disease of a much more extended range, that we can often follow the life-history of our patients from the cradle to the grave." The family practitioner, in short, has opportunities which neither the hospital physician nor the narrower specialist ever has, of seeing individuals in their entirety, from youth to age; and not only so, but of seeing families in their entirety, and of watching the facts of heredity in full and daily operation. He of all others is in the natural position which the medical philosopher should occupy. The specialist or the pure consultant is a mere occasional person, and must of necessity take an occasional view. It is true he has unique opportunities of studying one or more forms of disease. But, after all, the real object of the doctor's care is the individual in whom disease manifests itself—the whole man, his constitution, his inherited peculiarities, his acquired abnormalities, his responsiveness to peculiar diets, drugs, atmospheres, and the like. All this is known to the family physician—known to him fully, intelligently, instinctively; and known to him alone. Given the philosophic faculty, the family physician inevitably becomes the medical philosopher. A philosopher of this kind the specialist can never become, except by a concourse of circumstances which is so rare as to be practically miraculous.

"Life in a normal state," says Dr. Campbell Pope, should be pleasant and easy, that is, not obtrusive on the consciousness; disease or uneasiness shows that something is wrong or abnormal, and may thus be defined as 'abnormal life.'" "Pleasant and easy"—the very words give a little hopeful thrill to the consciousness. This, then, is what the modern physician aims at, when he happens to be a philosopher and a lover of his kind as well as a physician. It is what Sir Andrew Clark would call a "noble ambition," a "worthy end." The physician, whether of the body or of the mind, is, by his very nature, an ameliorator. His business is to make life more tolerable, more hopeful, more successful, more pleasant. But in order to do this he must begin at the beginning; he must understand his human watch before he can regulate it. He must not be a mere wearer or winder-up of the chronometer, he must be a maker; or, since that is not possible with the human watch, he must thoroughly understand the internal cosmogony, and the external environment, and learn to touch the delicate wheels and springs with the sure hand of the competent and sympathetic expert.

If disease be abnormal life, what is life itself? "Life," says Dr. Campbell Pope, "is the result of a series of interactions between an organism and its environment." We need not quarrel with the definition. It may be expressed in another way by saying that a man is what he is by virtue of his natural constitution and his general surroundings. From a "beginnings of disease" point of view we want to know always what a particular human being was as to his constitution and inherited peculiarities when he was born; and what sort of constitution-modifying surroundings he has been subjected to during his lifetime. We must make a little effort to understand the almost bewildering complexity of life as it manifests itself in the human being, compared with what may be as truly called "life" in the protoplasmic and practically undifferentiated amœba. "The life which we, as medical practitioners, have to study is not so simple as that of the amœba floating in the drop of water. It is a life of infinitely more complicated structure, consisting not of one cell, but of millions, arranged into specialised organs and tissues, carrying out functions subservient to the life of the whole structure: each cell, and each organ and tissue having its own special environment, and the whole structure surrounded by an environment complex beyond description, and differing for every individual."

Disease, when divested of the pain with which it is associated, and of the sympathy which it evokes, is in ultimate analysis—"merely an abnormality." Most true; but when your very "norms" are so profoundly mysterious as in many cases to altogether elude detection, who shall give a clear and convincing account of your still more mysterious abnormalities? The normal physiology of the human body is in many profound particulars imperfectly understood. What, then, are the possibilities of knowledge when abnormalities and pathologies are the subjects of investigation? Dr. Campbell Pope tells us very clearly and convincingly what we already know; he does not tell us what we do not know, but what we are most anxious to discover.

In a general way, however, Dr. Pope addresses to both doctors and patients much-needed words of counsel. Environment is the keystone of his position. What a man owns by inheritance in physiology, he owns it whether he will or will not. It is there within him, and he must make the best he can of it. But environment is much more amenable to modification. Environment is so all-powerful that it can in due time modify even inherited and inhering peculiarities of strength or weakness. "All disease begins mediately or immediately in the environment," says a recent Cavendish lecturer. "The environment is in the last resort the sole cause of disease," says another learned medical author. These generalisations require to be philosophically comprehended, and when they are so comprehended they are seen to be true for all practical purposes. The martyr to catarrhs is generally the victim of his environment. The sufferer from rheums and neuralgias suffers because of his environment. The names of many diseases instantly connote environment to the medical mind. Take, for example, pleurisy, bronchitis, pneumonia, consumption, peritonitis, nephritis, hepatitis, and the far less formidable but more common headache, dyspepsia, neuresthenia, and a host of other troubles which are as natural to some human beings as the sight of the sun in the morning or of the winter's snow.

What is the practical outcome of it all? The answer is obvious. Since environment is the keystone of healthy, happy, and successful life, let doctors study environment; let patients also, and even more, devote their best intelligence to the practical aspects of environment. Dwell in a pure atmosphere. This is the first commandment of physiology. Eat and drink what is serviceable for health, and nothing else. This is the second. Work strenuously, but not longer nor harder than your strength warrants. Live cleanly and sleep soundly. Such advice as this is as needful for the doctor as for the patient: almost more needful indeed, because the patient trusts the doctor as a master of the knowledge of environment, and takes his guidance from him. What is, above all others, the one aim of the practising physician? Is it not the putting of his patient into such internal and external environment as will enable the recuperative power within to have full and unhindered opportunity of exerting itself? The renewal of life must come from within. That renewal the physician may stimulate; but his chief function, when all is said, is the removal of impedimenta. "Environment," let us repeat, "is the keystone of disease, and it is also the keystone of cure."

